# Like Father, like Child: Early Life Family Adversity and Children’s Bullying Behaviors in Elementary School

**DOI:** 10.1007/s10802-017-0380-8

**Published:** 2017-12-19

**Authors:** Else E. de Vries, Marina Verlinden, Jolien Rijlaarsdam, Vincent W. V. Jaddoe, Frank C. Verhulst, Louise Arseneault, Henning Tiemeier

**Affiliations:** 1 The Generation R Study Group, Erasmus MC - University Medical Center, Rotterdam, The Netherlands; 2 Centre for Child and Family Studies, Leiden University, Leiden, The Netherlands; 3 Department of Child and Adolescent Psychiatry, Erasmus MC - Sophia Children’s Hospital, University Medical Center, Rotterdam, The Netherlands; 4 Department of Pediatrics, Erasmus MC - University Medical Center, Rotterdam, The Netherlands; 5 Department of Epidemiology, Erasmus MC - University Medical Center, Rotterdam, The Netherlands; 6 Medical Research Council Social, Genetic and Developmental Psychiatry Centre, Institute of Psychiatry, King’s College, London, UK; 7 Department of Psychiatry, Erasmus MC - University Medical Center, Rotterdam, The Netherlands

**Keywords:** Bullying, Family adversity, Hostility, Harsh parenting, Discipline, Family distress

## Abstract

Family adversity has been associated with children’s bullying behaviors. The evidence is, however, dominated by mothers’ perceptions of the family environment and a focus on mothers’ behaviors. This prospective population-based study examined whether children’s bullying behaviors were associated with mother- and father-reported family adversity, assessed before and after child birth. Peer-nominations were used to assess bullying behaviors of 1298 children in elementary school (mean age 7.5 years). The following paternal risk factors were prospectively associated with children’s bullying behaviors: (1) father-reported prenatal family distress, (2) fathers’ hostility at preschool age, and (3) fathers’ harsh disciplinary practices at preschool age, but effect sizes were relatively small. The effect of maternal risk factors was less consistent, only mother-reported family distress in childhood was associated with children’s bullying behaviors. The associations were independent of background family risk factors (i.e., life stress, contextual factors, and other background factors such as parental education and risk taking record) and early childhood externalizing problems. Moreover, our results indicated that father-reported family adversity predicted children’s bullying behaviors over and above the background family risk factors, early childhood externalizing problems and mother-reported family adversity. We also demonstrated that the association of fathers’ prenatal hostility and family distress with subsequent bullying behavior of their child at school was partly mediated by fathers’ harsh disciplinary practices at preschool age. Our findings highlight the importance of fathers’ behaviors in the development of children’s bullying behaviors.

**Electronic supplementary material:**

The online version of this article (10.1007/s10802-017-0380-8) contains supplementary material, which is available to authorized users.

## ᅟ

Bullying is a widespread problem during school years, with about one-third of children being involved in bullying in early elementary school (Jansen et al. 2012b). School bullying is typically defined as intentional and repeated peer aggression, both physically and psychologically, causing a power imbalance between a bully and a victim (Olweus 1993). Both children who bully and those who are the victims of bullying have more behavioral and emotional problems and tend to perform less well at school than children who are not involved in bullying (Sourander et al. 2007; Arseneault et al. 2010). Numerous studies have identified correlates of familial risk factors and children’s bullying behaviors at school. However, the evidence is dominated by mothers’ perceptions of the family environment and mothers’ behaviors. Examining the role of fathers, next to mothers, is key for understanding early risk factors within the family that may predispose children to engage in bullying behavior and, ultimately, for the effective prevention of school bullying. Therefore, the current prospective population-based study aimed to clarify whether children’s bullying behaviors are associated with paternal and maternal family risk factors, assessed before and after children were born.

The social cognitive theory (first known as social learning theory; Bandura 1978, 1986) provides a rationale for the link between children’s bullying behaviors, interpreted as an aggression phenomenon, and family risk factors. This theory posits that behavior is determined by the dynamic interaction between the social environment (such as witnessing parents’ behaviors) and internal factors (such as feelings, beliefs and expectations). Based on social cognitive theory, family-relational schema suggests that children develop beliefs and expectations from family experiences about what happens during conflictual situations that arise in close relationships (Perry et al. 2001). These internal representations about expected patterns of interactions lead children to misinterpret (social) cues, and respond more aggressively in new or conflictual situations. Through observational learning, children also develop beliefs about the likelihood of (positive) outcomes that result from aggressive behaviors. For example, when children observe an aggressive parent they develop the expectation that behaving aggressively has the benefit of getting attention or getting your way. Further, children might learn self-serving beliefs from their parents about behaving harmfully towards others without experiencing remorseful feelings afterwards, i.e., they morally disengage (Bandura 1999). Another aspect of the social cognitive theory is self-efficacy, which implies beliefs about one’s capabilities to perform the behaviors that are required to reach the desired outcome (Bandura 1986). For example, children from aggressive parents might be more confident in asserting themselves or to act aggressively. Thus, growing up in an adverse family environment predisposes children to develop behavioral and social-cognitive problems that may contribute to school bullying.

In their meta-analyses, Lereya et al. (2013) concluded that negative parenting behavior constitutes a risk factor for becoming involved in bullying. However, the vast majority of bullying research did not distinguish between the roles of fathers and mothers (e.g., Copeland et al. 2013; Schwartz et al. 1997; Burk et al. 2008). This has occurred despite research documenting the important role of fathers in child development (Lamb 2004; Ramchandani and Psychogiou 2009). Aggressive and antisocial behavior of fathers, and less that of mothers, has consistently been associated with aggressive behavior of their child (Avakame 1998; Jaffee et al. 2003; Stover et al. 2016). Moreover, fathers’ hostility in particular is strongly associated with children’s externalizing and aggressive behavior (Carrère and Bowie 2012; Stover et al. 2016). To investigate the specific role of fathers, next to mothers, in the development of children’s bullying behaviors, the current prospective population-based study utilized parents’ own perceptions of the environment and also their behaviors. In this study, we defined family adversity as consisting of factors that contribute to an adverse family environment: parental hostility, family distress, and harsh disciplinary practices. Thus, we defined family adversity as consisting of both traits and behaviors of parents. In line with the reasoning of Cantor (1990) and McAdams (1995), global traits of parents (characteristics that they “have”, such as hostility) are as vitally important as more contextualized behaviors (their “doing”, such as harsh parenting). By studying traits and specific behaviors side-by-side, we obtained a more thorough picture of the various kinds of adversities children face in the family environment.

### Previous Research on Family Adversity and Children’s Bullying Behaviors

Parental hostility poses a significant threat to child development (Richmond and Stocker 2008). Hostile behavior of parents (e.g., irritability, uncontrollable outbursts of temper) is directly associated with children’s externalizing and aggression problems (Carrère and Bowie 2012; Rijlaarsdam et al. 2014; Stover et al. 2016). However, to our knowledge, little research has been conducted that specifically examined the effect of having hostile parents in relation to bullying behavior of the child. Several studies examined hostile parenting as a risk factor of child bullying behavior (e.g., Schwartz et al. 1997), but this concerns parenting practices as opposed to the effect of hostility symptoms of parents. One might argue that hostile symptoms are manifested in the same way as hostile parenting practices. However, hostile symptoms of parents (including both state and trait hostility) could affect children through other mechanisms than only parent-child interactions. Parental hostile interpretations and hostile behaviors across different situations may serve as a model for children’s developing ideas about the world and result in more general hostile expectations and beliefs (Bandura 1978, 1986; Perry et al. 2001). Therefore, we focused on the broader concept of hostile symptoms of parents. Because parental hostility has been related to externalizing problems, we posit that parental hostility predisposes children to bullying behavior.

The partner relationship is often affected by parental psychopathology, such as hostility (Gordis et al. 2001). Children who bully are often exposed to family conflict, and their families often lack cohesion (Bowes et al. 2009; van Hoof et al. 2008). Typically, bullies also experience more family distress (Copeland et al. 2013; Burk et al. 2008). Therefore, children in these families are not only at an increased risk of developing behavior problems because they have a parent with psychological problems, but possibly also due to exposure to family distress. The studies just mentioned, however, can be prone to reverse causality as most of these studies did not correct for baseline levels of child behavior problems and did not examine family distress before the child was born.

Parents are more likely to endorse harsh disciplinary practices when they experience family distress or symptoms of hostility by spilling over this negativity into parent-child interactions (Frias-Armenta and McCloskey 1998; Jansen et al. 2012a; Stover et al. 2016). Importantly, harsh disciplinary practices like yelling and threatening have repeatedly been related to externalizing and aggressive behavior in children (e.g., Chang et al. 2003; Lereya et al. 2013; Pinquart 2017). Children who bully report that support from their parents is lacking (Demaray and Malecki 2003). Conversely, their parents report more use of punishment (Stevens et al. 2002). In a single-informant study, adolescents’ report of being more harshly disciplined by their parents mediated the association between fathers’ and mothers’ parenting styles (such as experiencing less support and understanding from their parents) and their own bullying behaviors (Gómez-Ortiz et al. 2016). In the current multi-informant study, we examined the association between fathers’ and mothers’ harsh disciplinary practices and children’s bullying behaviors. Moreover, we explored whether prenatal family distress or prenatal hostility is spilled over into parent-child interactions by the use of harsh disciplinary practices, eventually leading to child bullying behavior.

### Father-Reported Family Adversity and Children’s Bullying Behaviors

The studies summarized above indicate that family adversity in general is associated with bullying behavior of the child. For several reasons, we hypothesized that adverse family characteristics of the father predict child bullying behavior independently of maternal family adversity. First, the manifestation of fathers’ hostility may have a unique quality accounting for the link between fathers’ hostility and children’s bullying behaviors. It is possible that fathers and mothers experience equal levels of hostile feelings, but mothers may internalize these feelings, whereas fathers may act them out. A possible explanation for this gender difference is that women, consistent with traditional gender roles, are socialized to internalize hostile feelings (Eagly et al. 2000). Given that children can develop behavioral and social-cognitive problems from observing (Bandura 1978, 1986; Perry et al. 2001), children may learn disruptive behavioral patterns mostly by observing the father. Moreover, when father’s hostile feelings are directed outwards, this may increase negativity in the family and further predisposes a child to bullying behavior.

Second, differences between the content of mother-child interactions and father-child interactions may explain the strong associations of father’s hostility and child behavior. Mothers are most often the primary caregivers for children regardless of employment status (Schoppe-Sullivan et al. 2013). Mothers are more involved than fathers in socialization (e.g., taking the child to social events), didactic (e.g., reading with the child), and caregiving (e.g., assisting the child with eating); whereas fathers are more involved in physical play (e.g., playing outside with the child) (Schoppe-Sullivan et al. 2013). Given that mothers are more involved in the child’s life, mother’s hostility or harshness might be compensated by the caring aspects of her parenting. Father’s involvement in physical play encourages obedience and the ability to deal with conflict situations with peers in a socialized manner (Paquette 2004). When the father is hostile and harsh, this hostility and harshness might be more visible and displayed more directly towards the child during the typical father-child interactions. For the child, this might result in learning inappropriate (yet perceived as effective) aggressive ways of resolving a conflict (Bandura 1978, 1986). Furthermore, consistent with the fathering vulnerability hypothesis (Cummings et al. 2004), there is evidence that fathers are more likely to spillover negativity from the marital relationship to the father-child relationship than mothers (Schofield et al. 2009; Stover et al. 2016). Hence, in this way, the child may be more exposed to hostile and harsh behaviors on behalf of the father rather than the mother.

Third, compared to mothers, fathers may respond differently to children’s temperament. For example, in response to their child’s display of emotions, fathers are often more punitive or dismissive, whereas mothers are more likely to encourage the child to express his or her feelings (Cassano et al. 2007). The harsh or hostile responses from the father, in turn, increase the likelihood that the child will react aggressively and bully other children (Lereya et al. 2013) through the aforementioned learning process of observing adverse behavior (Bandura 1978, 1986).

### Current Study

This study will focus specifically on father-reported family adversity because of its presumed salience to the development of children’s bullying behaviors. In addition, we also examine four important aspects that have not been tested yet. First, the direction of the association between family adversity and children’s bullying behaviors is unclear. In our study, parental hostility and family distress were assessed twice, including once before the birth of the child, which allowed us to examine whether these family adversities are precursors of children’s bullying behaviors. Additionally, studying exposure to harsh disciplinary practices at preschool age, prior to school bullying, can reveal important information about the role of (harsh) parenting in later bullying. However, the association between postnatal family adversity and children’s bullying behaviors may be bidirectional as children’s behavior problems can influence disciplinary practices or increase the distress within families (Pardini 2008; Patterson 1982). In order to address alternative directions of association, we adjusted our analyses for child externalizing behavior as reported by the mother when the child was 18 months old.

Second, when the same informant reports on both the risk factors and on bullying involvement, the associations between family adversity and children’s bullying behaviors might be inflated by common method variance. To prevent this problem, we used both parents and children’s classmates as informants. Moreover, by using a peer nomination method that relies on ratings by classmates to assess bullying involvement, we avoided the problem that self-reported bullying involvement data are often biased by social desirability (Griffin and Gross 2004).

Third, adversity of the family environment involves a range of other background family risk factors that often co-occur, such as stressful life events, financial or housing problems (Appleyard et al. 2005). Without consideration of these background family risk factors, alternative explanations remain plausible for associations between family adversity and children’s disruptive behaviors (Davies et al. 2006). Background family risk factors can strain the family system and interfere with the parents’ ability to sensitively care for their child (e.g., less time to spend with the child, or aversive parenting behaviors) (Ramchandani and Psychogiou 2009; Ostberg and Hagekull 2000). Therefore, we examined whether background family risk factors are associated with later bullying behaviors of children. Subsequently, we examined whether the association between family adversity and children’s bullying behaviors changed when accounting for background family risk factors.

Fourth, in addition to parental gender differences, child gender differences should also be taken into account as fathers and mothers may differentially impact bullying behaviors in their sons and daughters. Boys are at higher risk for being involved in bullying than girls (Jansen et al. 2012b), which could be a result of differential socialization processes by parents (Keenan and Shaw 1997; Eagly et al. 2000). We therefore examined whether child gender moderated the association between family adversity and children’s bullying behaviors.

In sum, the objective of our study was to examine the association of separate father-reported family adversity factors, i.e., hostility, family distress, and harsh disciplinary practices, assessed pre- and postnatally, in relation to children’s bullying behaviors in early elementary school. These associations were contrasted with those between mother-reported family adversity and children’s bullying behaviors. We studied these objectives in a large prospective population-based birth cohort while accounting for possible influences of various socio-demographic factors, background family risk factors, and child externalizing problems. The first hypothesis we tested was that the contribution of father-reported family adversity to children’s bullying behaviors is more pronounced than mother-reported family adversity. The second hypothesis we tested was that the association between prenatal family adversity and children’s bullying behaviors is partly mediated by parental harsh disciplinary practices. Importantly, the current study assessed family adversity before and after children were born. This enabled us to examine the timing of the contribution of family adversity to children’s bullying behaviors. Moreover, by using a peer nomination method to assess school bullying, we minimized potential biases of common method variance and social desirability.

## Method

### Design and Study Population

This study is embedded in the Generation R Study (Jaddoe et al. 2012), a large population-based cohort of children followed from fetal life onwards. The initial cohort comprised 9778 pregnant women with an expected delivery date between April 2002 and January 2006, living in Rotterdam, the Netherlands. The response at baseline was 61%, and general follow-up rates until the age of 6 years exceed 80%. Regular extensive assessments have been carried out among children and parents (see for detailed description Tiemeier et al. 2012). Written consent was obtained from the parents. The study has been approved by the Medical Ethics Committee of the Erasmus Medical Center.

Bullying was assessed in a subsample of Generation R Study participants. An assessment of bullying was carried out in a study of peer relations across 37 elementary schools (190 school classes) in Rotterdam and suburbs, at the time when the oldest Generation R participants attended grades 1–2 of elementary school (Verlinden et al. 2014). In total, 4017 children from the participating schools in Rotterdam completed assessment. Out of these 4017 children, 1664 children were Generation R Study participants. Parents of 1590 Generation R participants provided consent for data linkage. Subsequently, peer reports of bullying was available for 1590 Generation R Study participants. The analyses for the present study were conducted in 1298 children with data on family adversity.

### Bullying Behaviors

Bullying behaviors were assessed in elementary school children in grades 1–2 (mean age 7.53 years, *SD* = 9.24 months) using the PEERS Measure (Verlinden et al. 2014). This interactive computerized instrument is a reliable and age-appropriate method of using peer nominations with young children.

All children in a class completed a computerized peer nomination assessment through which they nominated those classmates, who bullied them. Bullying was explained to children as intentional, repeated and continuous actions of peer aggression and that the victim finds it difficult to defend him or herself (Olweus 1993). Questions were accompanied by audio instructions and visual illustrations (Verlinden et al. 2014). Four different forms of bullying were assessed by a yes/no question: (1) physical bullying (e.g., kicking or pushing); (2) verbal bullying (e.g., calling names); (3) relational bullying (e.g., excluding); and (4) material bullying (e.g., taking away or hiding belongings). If a question was answered affirmatively, children were asked to nominate those classmates who bullied them. To perform the nominations, they could click on the photos of the classmates on the screen.

The nominations a child received from classmates were used to calculate individual bullying scores. The received nominations were weighted by the number of classmates performing the evaluation. Thus, the individual bullying scores were based on the ratings by about 20 classmates with regards to each bullying question, and represent the extent to which a child was perceived as a bully by his or her classmates. The scores on the four different forms of bullying were averaged, with higher scores representing more bullying nominations by classmates.

### Family Adversity Factors

#### Parental Hostility

Hostility symptoms of fathers and mothers were assessed prenatally during pregnancy (at 20 weeks) and again postnatally when the child was 3 years old. We used the Hostility subscale of the Dutch version of the Brief Symptom Inventory (BSI; de Beurs 2004; Derogatis 1993), a validated self-report questionnaire to ascertain psychological symptoms (both state and trait; Suris et al. 2004) of individuals. The Hostility subscale consists of 5 items (“Easily becoming bored or feeling irritable”, “Uncontrollable bursts of anger”, “An urge to hit, injure or cause pain to others”, “An urge to damage or break things”, “Often getting involved in arguments”). Fathers and mothers reported the extent to which each item described their feelings in the past week, using answer categories ranging from “not at all” (0) to “extremely” (4). Item scores were summed and divided by the amount of completed items. Higher scores represent more hostility. The internal consistencies were *α* = .70 and *α* = .62 for fathers’ prenatal and postnatal ratings respectively*,* and *α* = .74 and *α* = .60 for mothers’ prenatal and postnatal ratings respectively.

#### Family Distress

Family distress as perceived by the parents was assessed with 12 items from the General Functioning Scale of the McMasters Family Assessment Device (Byles et al. 1988). Fathers and mothers filled this questionnaire during pregnancy (at 20 weeks). Mothers, but not fathers, completed it again when children were 6 years old; we used this measure as a postnatal measure of family distress. Half of the items describe healthy functioning (e.g., “In times of crisis we can turn to each other for support”); the other half portrays unhealthy functioning (e.g., “We avoid discussing our fears and concerns”). Parents rated how well each item described their family by selecting a response ranging from “strongly agree” (1) to “strongly disagree” (4). The items describing unhealthy functioning were reverse coded. The scores per item were summed and dived by 12 yielding a total score ranging from 1 to 4. A higher total score represents less well-functioning families or more family distress. The internal consistency of family distress, measured by Cronbach’s alpha, was .88 (father-reported scales) and .90 (mother-reported scales).

#### Harsh Disciplinary Practices

To obtain information about parental disciplinary practices, fathers and mothers each filled in an adapted version consisting of 10 items of the Parent-Child Conflict Tactics Scale (CTS-PC; Straus et al. 1998) when the child was 3 years old (Jansen et al. 2012a). The review board of the Generation R Study decided to exclude three items on hitting and spanking because some forms of harsh punishment encompass possibly illegal practices in the Netherlands. Additionally, one question about throwing the child out of the house was excluded to make the assessment more age appropriate. Parents rated their use of disciplinary practices (e.g., “I shouted or screamed angrily at my child”, “I gave my child a good shake”) during the past 2 weeks on a 6-point scale ranging from “never” to “five times or more”. Several categories were combined due to low prevalence rates, resulting in three categories: “never” (0), “once” (1), and “twice or more” (2). Item scores were summed and divided by the amount of completed items. Higher scores reflect higher incidence of harsh discipline. The internal consistency of this 6-item construct was *α* = .51 for fathers and *α* = .56 for mothers. These relatively low reliabilities can most probably be explained by the large number of zero responses in our population-based sample.

### Background Family Risk Factors

Following a method described elsewhere (Cecil et al. 2014; Rijlaarsdam et al. 2016), we created scores for three domains that can be considered background family risk factors. We used father’s and mother’s prenatal reports to gather information about these three domains: (i) life stress (e.g., death in family, work problems), (ii) contextual factors (e.g., financial difficulties, housing problems), and (iii) other background factors (e.g., educational level, criminal involvement, substance abuse). For each domain, items were summed and divided by the number of completed items. Higher scores correspond to the presence of more background family risk factors in the respective domain. See Supplementary Table 1 for full item descriptions.

### Covariates

Based on previous studies of bullying, we adjusted our analyses for the following socio-demographic and psychosocial covariates as they were considered possible confounding factors in the association between family adversity and school bullying: child age, gender, national origin, monthly household income and parity (Jansen et al. 2012b; Shetgiri et al. 2014), and child pre-existing externalizing problems (Bowes et al. 2009; Cecil et al. 2014). We considered financial difficulties as a background family risk factor (falling under the contextual factors domain) and household income as a possible confounding factor. In the Netherlands, an extended social security system provides financial security when ones income is insufficient due to, for example, unemployment. As a result, experiencing financial difficulties is a better indicator of stress, whereas a low income in the Netherlands does not necessarily imply financial difficulties but is a broad occupational, social and educational indicator.

Information about child gender and date of birth was obtained from midwife and hospital registries. All other socio-demographic covariates were assessed by prenatal questionnaires. National origin was defined by country of birth of the parents and categorized as Dutch, other Western and non-Western. Birth order of the child (i.e., parity) ranged from “No older siblings” to “Three or more older siblings”. Net monthly household income was categorized into “Less than €1,200” (below social security level), “€1,200 to €2,000” (modal income), and “More than €2,000” (above modal income). Marital status was assessed by questionnaire and defined as single during pregnancy or when the child was 3 years old.

Children’s externalizing problems at 18 months were assessed by mother report on the 24-item externalizing scale of the Child Behavior Checklist for toddlers, CBCL/1½–5 (Achenbach and Rescorla 2000). The psychometric properties of the CBCL are well established (Achenbach and Rescorla 2000). The mother evaluated child behavior (e.g., “Cannot sit still, is restless or hyperactive”, “Fights a lot”) over the past two months using answer categories ranging from “not true” (0) to “very true or often true” (2). Higher scores represent more externalizing problems. The internal consistency for the externalizing scale was *α* = .88.

### Statistical Analyses

Baseline characteristics of the data were explored and Spearman’s correlation coefficients between all family adversity variables and children’s bullying behaviors were calculated. To investigate possible selection bias (non-response analysis), we compared differences in baseline characteristics of participants with (*n* = 1298) and without (*n* = 292) data on family adversity with the Chi-square statistic, one-way ANOVAs, and the Mann-Whitney *U* test. Likewise, we compared baseline characteristics between boys (*n =* 631) and girls (*n =* 667).

For our main analyses, to approximate a normal distribution, the background family risk factors, hostility, bullying and externalizing scores were square root transformed; the family distress and harsh parenting scores were logarithmic (Log 10) transformed. Transformed variables were standardized to allow comparability.

To examine associations between father- and mother-reported family adversity (i.e., hostility, family distress, and harsh parenting) and children’s bullying behaviors (hypothesis 1), three multilevel linear regression models were conducted for each predictor separately. In order to examine the effect of the background family risk factors (i.e., life stress, contextual factors, and other background risks factors) and early childhood externalizing problems on children’s bullying behaviors, we also included these as predictors in the first two models. In model 1, unadjusted multilevel linear regression analyses were performed for each predictor separately. Next, to assess whether the associations were similar for boys and girls, we explored interactions between each predictor and child gender. Multiplicative interaction terms that were added to the model to test gender effect modification were non-significant (*p* > .05). In model 2, we adjusted for socio-demographic characteristics (i.e., child gender, age, ethnicity, parity, and monthly household income) by adding these variables as covariates. Since variables can be confounders without empirical evidence for it in the current dataset (Lee 2014), we based our choice of confounders on previous studies and added them as covariates without backward or forward selection methods. In order to address alternative explanations and directions of the associations between family adversity and children’s bullying behaviors, in model 3 we additionally adjusted for the background family risk factors (i.e., life stress, contextual factors, and other background risks factors) as well as for early childhood externalizing problems. To test for differences between the effect estimates (i.e., standardized regression coefficients) of each specific father-reported family adversity factor and the corresponding mother-reported family adversity factor, we produced 84% confidence intervals around the effect estimates and examined the overlap. As reported by Julious (2004), the level of statistical significance between the two effect estimates would be .05 or lower if the 84% confidence intervals do not overlap.

In further analyses, in order to get an indication of whether father-reported family adversity predicts children’s bullying behaviors over and above that of the background family risk factors, early childhood behavior problems and mother-reported family adversity, we tested a hierarchical multilevel model in which the following predictors were entered cumulatively in four blocks: (1) background family risk factors, (2) early childhood externalizing problems, (3) mother-reported family adversity, and (4) father-reported family adversity. The cumulative blocks were compared using the chi-square likelihood ratio test.

Lastly, to examine whether the association between prenatal family adversity and children’s bullying behaviors is partly mediated by parental harsh disciplinary practices (hypothesis 2), we performed mediation analyses. We accounted for all socio-demographic covariates and background family risk factors. Additionally, when considering fathers’ prenatal hostility or family distress as a predictor, we adjusted the analysis for mothers’ prenatal hostility or family distress respectively, and vice versa. Likewise, the analyses regarding fathers’ harsh disciplinary practices as a mediator were adjusted for mothers’ harsh disciplinary practices, and vice versa. The bootstrapping method, a nonparametric resampling procedure, was used (Hayes 2013). With this method, a confidence interval around each estimate of the indirect (i.e., mediating) effect was computed. If zero is not included within the confidence interval, an indirect effect is present. As recommended by Wen and Fan (2015), the proportion of the indirect effect relative to the total effect was calculated as an effect size measure (P_M_). Using the SPSS script for the Indirect procedure (Hayes 2013), bootstrapping for five imputed datasets was performed separately; 5000 samples were requested; and 95% bias-corrected confidence intervals were calculated. Results from the five bootstrap analyses were averaged together.

Missing values of the covariates and risk factors were estimated using multiple imputation. The reported effect estimates are the pooled results of five imputed datasets. Results for our complete case sensitivity analysis were essentially unchanged. We accounted for the clustered data structure (i.e., children from the same school classes were tested) by performing multilevel analyses using school class as a grouping variable. For each predictor, the best fitting model (i.e., fixed effects model, random intercepts model, or random intercepts and slopes model) was chosen using the chi-square likelihood ratio test. For all predictors, the chi-square test of the difference in −2 log likelihood showed a significant increase in explained variance between the fixed effects model and the random intercepts model (*p* < .05), but not between the random intercepts model and random intercepts and slopes model (*p* > .05). Therefore, only results from the random intercepts models are reported. Statistical analyses were performed using SPSS Statistics 21.0 (IBM Corporation, Somers, NY, USA).

### Non-response Analyses

Data were more often missing for children with non-Western national origins (64% vs. 25%; *χ* ^*2*^ (2) = 145.65, *p* < .001), families with a lower income (44% vs. 12%; *χ* ^*2*^ (2) = 90.04, *p* < .001), and higher parity (11% vs. 2%; *χ* ^*2*^ (3) = 82.98, *p* < .001). Moreover, higher background family risk factors scores (mean rank 793 vs. 599; *U* = 21,736, *z* = −4.00, *p* < .001), higher scores of mothers’ prenatal hostility (mean rank 702 vs. 560; *U* = 23,027, *z* = −3.33, *p* = .001), higher scores of mother-reported family distress 6 years after birth (mean rank 746 vs. 611; *U* = 45,919, *z* = −3.68, *p* < .001), and higher scores of mothers’ harsh parenting (mean rank 681 vs. 528; *U* = 10,318, *z* = −2.66, *p* = .008) were found in participants with missing data.

## Results

Child and parent characteristics of the study sample are presented in Table 1. Our sample comprised 49% boys, 64% of children were of Dutch national origin (Table 1). Bullying behavior was assessed at the mean age of 7.5 years (*SD* = 9.2 months). In comparison with girls, boys displayed more externalizing behavior (mean rank 536 vs 477; *U* = 112,499, *z* = −3.21, *p* = .001), more bullying behavior (mean rank 750 vs. 555; *U =* 147,260, *z =* −9.37, *p <* .001), and were more harshly disciplined by their father (mean rank 471 vs. 391; *U* = 74,339, *z* = −4.88, *p* < .001) and mother (mean rank 553 vs. 485; *U* = 116,190, *z* = −3.75, *p* < .001). As shown in Table 2, all family adversity variables were significantly correlated with children’s bullying behaviors, with the exception of mothers’ hostility 3 years after birth of their child.

**Table 1:** Sample characteristics

	All children (*N* = 1,298)		Boys (*n* = 631)		Girls^b^ (*n* = 667)	
Characteristic	*n*	*M (SD)* ^a^	*n*	*M (SD)* ^a^	*n*	*M (SD)* ^a^
Child characteristics						
Gender (boys, %)	1,298	48.61				
Age (years)	1,298	7.52 (0.77)	631	7.55 (0.77)	667	7.50 (0.78)
Child national origin (%)						
Dutch	832	64.35	398	63.17	434	65.46
Other Western	144	11.14	71	11.27	73	11.01
Non- Western	317	24.52	161	25.56	156	23.53
Parental characteristics						
Monthly household income (prenatal) (%)						
Less than €1200 (below social security)	133	12.16	63	11.71	70	12.59
€1200 to €2000 (average)	194	17.73	110	20.45	84	15.11
More than €2000 (modal)	767	70.11	365	67.84	402	72.30
Marital status (% single)	1027	15.68	500	17.60	527	13.85
Older sibling(s) in family (parity >0) (%)	1264	43.67	615	43.25	649	44.07
Background family risk factors						
Life stress	1069	2.17 (1.61)	508	2.14 (1.59)	561	2.19 (1.63)
Contextual factors	969	0.97 (1.34)	464	1.00 (1.38)	505	0.95 (1.30)
Other background factors	713	0.36 (0.70)	346	0.37 (0.75)	367	0.35 (0.65)
Early childhood behavioral problems^c^						
Externalizing problems 18 months	1010	10.43 (6.66)	486	11.06 (6.58)	524	9.84 (6.68)*
Family adversity						
Hostility, prenatal^d^						
Father	921	0.17 (0.31)	452	0.18 (0.31)	469	0.16 (0.30)
Mother	1077	0.28 (0.40)	513	0.29 (0.43)	564	0.27 (0.37)
Family distress, prenatal^e^						
Father	920	1.53 (0.41)	450	1.53 (0.42)	470	1.52 (0.41)
Mother	1164	1.55 (0.47)	552	1.56 (0.47)	612	1.54 (0.46)
Hostility, 3 years after birth^d^						
Father	896	0.17 (0.27)	431	0.18 (0.28)	465	0.17 (0.28)
Mother	1031	0.18 (0.26)	503	0.18 (0.25)	528	0.18 (0.27)
Harsh discipline, 3 years after birth^f^						
Father	857	1.78 (1.74)	407	2.07 (1.84)	450	1.51 (1.61)*
Mother	1035	2.08 (1.87)	504	2.34 (2.06)	531	1.84 (1.63)*
Family distress, 6 years after birth^e^						
Mother	1140	1.51 (0.42)	551	1.53 (0.43)	589	1.49 (0.41)
Outcome						
Child bullying behavior^g^	1298	0.05 (0.05)	631	0.06 (0.06)	667	0.03 (0.03)*

Values are based on untransformed variables

^a^Unless otherwise indicated. ^b^Gender differences were tested with the chi-square statistic for categorical variables and the Mann-Whitney *U* test for non-normally distributed continuous variables. Significant gender difference (*p* < .05) are denoted by an asterisk. ^c^Child externalizing behavior problems were assessed with CBCL/1½-5, the Dutch version of the Child Behavior Checklist. ^d^Hostility symptoms were measured with the Brief Symptom Inventory. ^e^Family distress was measured with the McMasters Family Assessment Device. ^f^Harsh disciplinary practices were assessed using a questionnaire based on the Parent-Child Conflict Tactics Scale. ^g^Bullying behaviors were assessed at age 8 years using the PEERS Measure

**Table 2:** Bivariate correlations between family adversity, early childhood behavioral problems and children’s bullying behaviors (*N* = 1298)

Variable	1	2	3	4	5	6	7	8	9	10
Hostility, prenatal										
1. Father	–									
2. Mother	.18*	–								
Family distress, prenatal										
3. Father	.27*	.23*	–							
4. Mother	.20*	.25*	.45*	–						
Hostility, 3 years after birth										
5. Father	.34*	.13*	.22*	.16*	–					
6. Mother	.11*	.25*	.14*	.19*	.19*	–				
Harsh discipline, 3 years after birth										
7. Father	.17*	.07	.12*	.11*	.32*	.16*	–			
8. Mother	.11*	.16*	.14*	.16*	.09*	.30*	.42*	–		
Family distress, 6 years after birth										
9. Mother	.10*	.18*	.29*	.44*	.17*	.24*	.15*	.16*	–	
Early childhood behavioral problems										
10. Externalizing problems 18 months	.13*	.17*	.16*	.13*	.08*	.18*	.13*	.24*	.16*	–
Outcome										
11. Child bullying behavior	.10*	.10*	.17*	.13*	.07*	−.02	.12*	.10*	.15*	.09*

Values are based on untransformed variables and represent Spearman correlation coefficients.

*p < .05

### Family Adversity and Children’s Bullying Behaviors

The results of the multilevel regression analyses predicting children’s bullying behaviors in early elementary school from background family risk factors, early childhood externalizing problems, and several components of family adversity (i.e., parental hostility, family distress, and harsh disciplinary practices) are presented in Table 3. In unadjusted model 1, the background family risk factor domains of contextual factors and other background factors, as well as children’s externalizing behavior problems, were associated with children’s bullying behaviors (all *p* < .05). Moreover, almost all family adversities were associated with children’s bullying behaviors, with the exception of mothers’ prenatal and postnatal hostility (*p* > .05).

**Table 3:** Multilevel regression analyses predicting children’s bullying behaviors from background family risk factors, early childhood behavioral problems and family adversity (*N* = 1298)

	Bullying score					
	Unadjusted (Model 1)		Adjusted for socio-demographic covariates (Model 2)		Additionally adjusted for background family risk factors and early childhood behavioral problems (Model 3)	
Variable	*B*	95% CI	*B*	95% CI	*B*	95% CI
Background family risk factors						
Life stress	0.04	[−0.01, 0.09]	0.02	[−0.03, 0.07]	n.a.	
Contextual factors	0.10***	[0.05, 0.15]	0.03	[−0.02, 0.09]	n.a.	
Other background factors	0.16***	[0.08, 0.23]	0.12**	[0.04, 0.19]	n.a.	
Early childhood behavioral problems						
Externalizing problems 18 months	0.10***	[0.04, 0.15]	0.06*	[0.01, 0.11]	n.a.	
Family adversity						
Hostility, prenatal						
Father	0.08*	[0.01, 0.15]	0.06	[−0.01, 0.12]	0.03	[−0.03, 0.10]
Mother	0.05	[0.00, 0.10]	0.00	[−0.05, 0.05]	−0.04	[−0.10, 0.02]
Family distress, prenatal						
Father	0.14***	[0.09, 0.20]	0.10***	[0.05, 0.15]	0.08**	[0.02, 0.13]
Mother	0.11***	[0.06, 0.16]	0.04	[−0.01, 0.09]	0.03	[−0.02, 0.08]
Hostility, 3 years after birth						
Father	0.08**	[0.02, 0.13]	0.06*	[0.01, 0.11]	0.06*	[0.001, 0.11]
Mother	0.02	[−0.04, 0.08]	0.01	[−0.05, 0.06]	−0.01	[−0.08, 0.05]
Harsh discipline, 3 years after birth						
Father	0.11***	[0.06, 0.16]	0.06*	[0.01, 0.11]	0.06*	[0.01, 0.11]
Mother	0.08**	[0.02, 0.14]	0.02	[−0.03, 0.08]	0.00	[−0.07, 0.06]
Family distress, 6 years after birth						
Mother	0.13***	[0.08, 0.18]	0.09***	[0.04, 0.14]	0.07**	[0.02, 0.12]

Presented coefficient: standardized *B* derived from multilevel linear regression analyses (using transformed variables). Model 2 was adjusted for the following socio-demographic covariates: child gender, age, ethnicity, parity, and parental income. Model 3 was adjusted for the socio-demographic covariates and additionally for background family risk factors (i.e., life stress, contextual risks, and other background factors) and early childhood behavioral problems (i.e., children’s externalizing problems at 18 months). CI = confidence interval.

* *p* < .05. ** *p* < .01. *** *p* < .001

After adjusting for socio-demographic covariates in model 2, the other background factors domain and children’s externalizing behavior problems remained associated with children’s bullying behaviors (*p <* .05). Furthermore, several family adversities remained associated with children’s bullying behaviors. First, the analyses of family distress showed that family distress as experienced prenatally by fathers (*B* = 0.10, 95% CI = [0.05, 0.15], *p* < .001) and as experienced 6 years after birth by mothers (*B* = 0.09, 95% [CI = 0.04, 0.14], *p* < .001) were associated with children’s bullying behaviors. Second, hostility symptoms of fathers 3 years after birth of their child (*B* = 0.06, 95% [CI = 0.01, 0.11], *p* = .02) were associated with children’s bullying behaviors. Third, results indicated that fathers’ but not mothers’ harsh disciplinary practices when their child was 3 years old were associated with children’s bullying behaviors (fathers *B* = 0.06, 95% CI = [0.01, 0.11], *p* = .02; mothers *B* = 0.02, 95% CI = [−0.03, 0.08], *p* = .44).

To examine whether the associations between family adversity and children’s bullying behaviors changed when accounting for background family risk factors and early childhood behavior problems, we additionally adjusted for life stress, contextual factors, other background factors, and child externalizing behavior in model 3. The results showed that father-reported prenatal family distress, fathers’ hostility 3 years after birth of their child, fathers’ harsh disciplinary practices, and mother-reported family distress 6 years after birth of their child remained associated with children’s bullying behaviors (all *p* < .05). However, the effect estimates of the father-reported family adversity factors were not significantly different to the corresponding mother-reported family adversity factors, since the 84% confidence intervals of the effect estimates overlapped (data not shown).

### Father-Reported Family Adversity and Children’s Bullying Behaviors

The results from the hierarchical multilevel model, in which we tested four blocks of predictors cumulatively, are presented in Supplemental Table 2. The comparison of block 3 to block 4 shows that father-reported family adversity predicted children’s bullying behaviors over and above that of the background family risk factors, early childhood externalizing problems and mother-reported family adversity (*χ* ^*2*^ (4) = 14.93, *p* < .01).^1^

### Mediation by Parental Harsh Disciplinary Practices

**Fig. 1:**
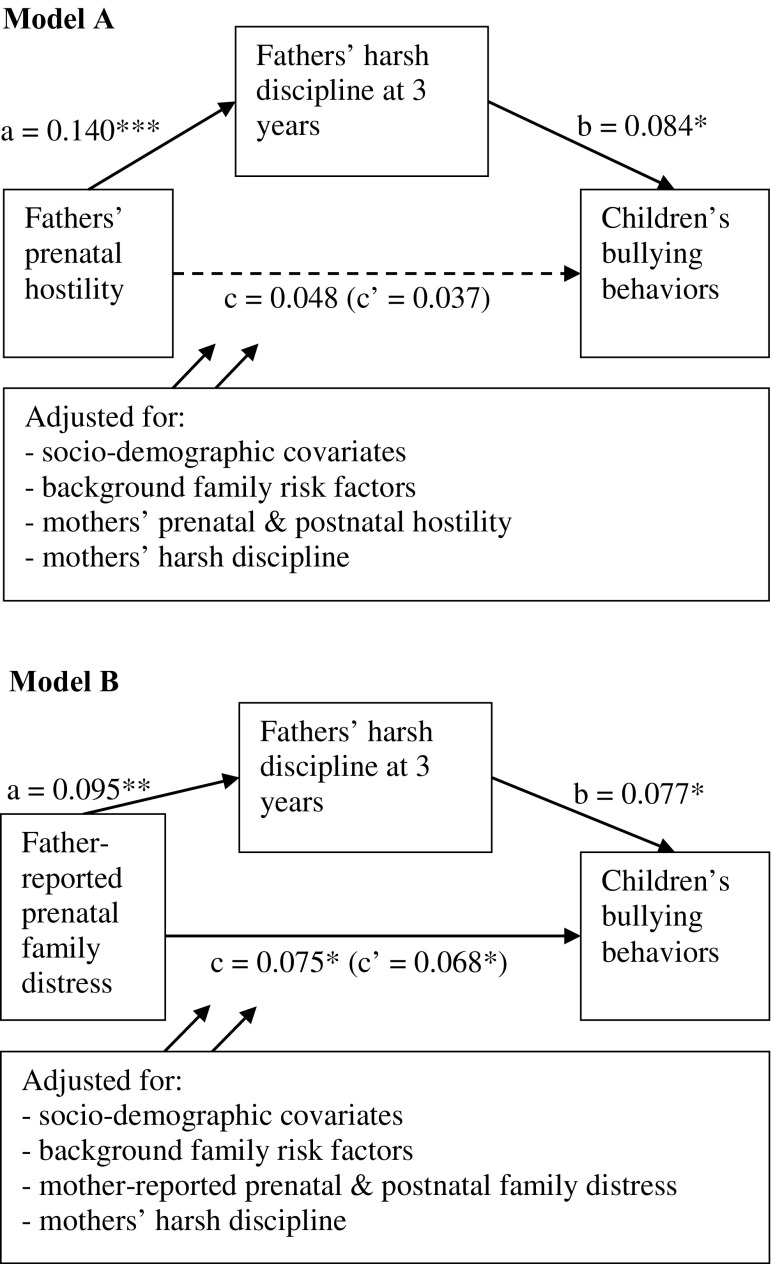
Mediation analyses (using transformed variables) predicting the impact of fathers’ prenatal hostility (model A) and father-reported prenatal family distress (model B) on children’s bullying behaviors in early elementary school via fathers’ harsh disciplining practices at 3 years. Values are standardized coefficient estimates. Models are adjusted for the socio-demographic covariates (child gender, age, ethnicity, parity, and parental income), background family risk factors (i.e., life stress, contextual factors, and other background factors), mothers’ harsh disciplinary practices, and for mothers’ prenatal and postnatal hostility (model A), or mother-reported prenatal and postnatal family distress (model B). *p < .05. **p < .01. ***p< .001

Several mediation models tested whether the association between prenatal family adversity and children’s bullying behaviors is partly mediated by harsh disciplinary practices. The association between prenatal hostility symptoms of fathers and children’s bullying behaviors was mediated through fathers’ harsh disciplinary practices (Fig. 1). The point estimate of this indirect effect was 0.012, with a 95% bias-corrected confidence interval from 0.004 to 0.023. Relative to the total effect (0.048), this gives an effect size of P_M_ = .25. In other words, 25% of the effect of fathers’ prenatal hostility on children’s bullying behaviors occurred indirectly through fathers’ harsh disciplinary practices. Likewise, the association between prenatal family distress as reported by fathers and children’s bullying behaviors was mediated through fathers’ harsh disciplinary practices. The point estimate of this indirect effect was 0.007, with a 95% bias-corrected confidence interval from 0.001 to 0.017. Relative to the total effect (0.075), this gives an effect size of P_M_ = .10, meaning that only a small proportion (10%) of the effect of father-reported prenatal family distress on children’s bullying behaviors occurred indirectly through fathers’ harsh disciplinary practices. The indirect effects of the models containing mothers’ prenatal hostility or family distress as predictors, however, were non-significant (results not shown).

### Sensitivity Analyses

Analyses presented in Table 3 were repeated in a subsample excluding single-mother families (i.e., single during pregnancy or when the child was 3 years old; Supplementary Table 3). The results gave a similar picture as in the full sample, although the association between fathers’ hostility 3 years after birth of their child and children’s bullying behaviors was slightly attenuated.

## Discussion

This study extends previous research by focusing on the specific role of fathers and mothers in the development of children’s bullying behaviors. Although the effect sizes were small, we showed that father-reported family adversity (consisting of both traits and behaviors) was prospectively associated with school bullying, whereas the effect of mother-reported family adversity was less pronounced. More specifically, when analyzing the family adversity factors separately, the following paternal risk factors were related to children’s bullying behaviors in early elementary school: (1) father-reported prenatal family distress, (2) fathers’ hostility at preschool age, and (3) fathers’ harsh disciplinary practices at preschool age. By contrast, only one family adversity factor reported by mothers was associated with children’s bullying behaviors, namely family distress in childhood. The associations were independent of background family risk factors (i.e., life stress, contextual factors, and other background factors such as parental education and risk taking record) and early childhood externalizing problems. In addition, our sensitivity analyses showed that the associations were similar for the subsample excluding single-mother families during pregnancy or when the child was 3 years old. Moreover, father-reported family adversity predicted children’s bullying behaviors over and above the background family risk factors, early childhood externalizing problems and mother-reported family adversity. However, when we compared the effect estimates of each father-reported family adversity factor to the corresponding mother-reported family adversity factor, we did not find a significant difference. Yet, taking all results into consideration, it is still striking that father’s behavior is consistently associated with later child bullying behavior.

The current study also sought to uncover the process by which prenatal family distress or prenatal hostility leads to children’s bullying behaviors at school by examining the mediational role of harsh disciplinary practices at preschool age. In line with our second hypothesis, we demonstrated that the association between prenatal family distress and prenatal hostility symptoms as reported by fathers and children’s bullying behaviors is partly mediated through fathers’ harsh disciplinary practices. The directionality of these finding are consistent with the main principle of the spillover hypothesis that negativity within the family undermines the ability of the parents to sensitively respond to their children (Erel and Burman 1995). These negative parenting behaviors, in turn, predict children’s bullying behaviors (Lereya et al. 2013).

It is important to interpret the current findings with the relatively small size of effects in mind. There are several reasons why the effect sizes were small and the effects were not very different for mothers and fathers. First, obtaining data from questionnaires might not be nuanced enough to detect the full spectrum of family adversity risk factors of fathers and mothers, leading to an underestimation of the effect sizes of our results. Second, we carefully avoided inflated effect estimates using different informants (i.e., fathers, mothers and peers) reporting on the risk factors and on bullying involvement. Third, with a longitudinal design large effect sizes should not be expected (Adachi and Willoughby 2015), especially when the interval between the assessment of the parental risk factors and the behavioral outcome in children is long (i.e., up to 7.5 years). Fourth, in comparison to a population-based study, effect sizes would probably be larger in at-risk or clinical samples (e.g., children from abusive families). Effect sizes do not have to be large to be relevant for public health (Rutledge and Loh 2004). Moreover, our results do point to a pattern that father’s behavior is consistently associated with later child bullying behavior, whereas this is not observed in mothers.

The development of disruptive behavior problems like bullying is influenced by a complex interplay between social, psychological, and biological factors (Sroufe 2009). The relative importance of these influences is unclear because genetic and environmental factors are confounded within families. For example, aggressive parents could have aggressive children as a result of genetic transmission, harsh disciplinary practices, or both. Thus, the negative effect of growing up in an adverse family environment on school bullying requires understanding social, psychological, and biological pathways.

Our findings are in line with the social cognitive theory (Bandura 1978, 1986) and the family relational schema (Perry et al. 2001). Given their salience for children, family distress, and more specifically fathers’ hostility and harshness are likely to influence children’s developing understanding of how to manage conflictual situations. Thus, children learn by observing their fathers that behaving in a hostile or harsh manner towards others is acceptable.

Children growing up in a disadvantaged socioeconomic environment experience more family adversity such as harsh disciplinary practices than children growing up in an advantaged socioeconomic environment (Bradley and Corwyn 2002). Economic distress in adoptive parents has been associated with harsh parenting and with child aggression (Stover et al. 2012), indicating that this chain of events is not simply attributable to genes shared by parents and children. Previously we found in a different sample that children from socioeconomically disadvantaged families have a particularly high risk of involvement in school bullying (Jansen et al. 2012b). Our finding that several father-reported family adversities are associated with bullying behaviors could therefore be confounded by socioeconomic disadvantage. However, we carefully accounted for socioeconomic status and it is not likely that the association between father-reported family adversity and children’s bullying behaviors can be mainly attributed to socioeconomic disadvantage.

Theories that focus on different developmental periods suggest that the preschool years, during which we measured parental hostility and harsh disciplinary practices, may be a sensitive period of heightened vulnerability to family aggression (Davies et al. 2006). Experiencing hostility or conflict between the parents or being harshly treated can have a special impact during early childhood as the capacity for emotional regulation is just beginning to emerge (Yates et al. 2003). Following emotional security theory (Davies et al. 2006), children’s emotional reactivity to parental hostility or conflict is accompanied by hypervigilance and arousal, which in turn increases behavior dysregulation (e.g., oppositionality) by amplifying children’s tendencies to scan and respond to potential danger. These biases to focus on threatening aspects of other’s actions can make the child more likely to respond to social challenges in an aggressive manner (Bascoe et al. 2009). Therefore, our findings that exposure to family distress, as well as fathers’ hostility and harsh disciplinary practices at preschool age, predicts later bullying behaviors, can in part be explained by the notion that family adversity causes patterns of emotion regulation and behavioral expression of the child that makes the child more likely to react with bullying behaviors towards peers.

Several biological mechanisms may underlie the observed association between family adversity and child bullying, which are not necessarily exclusive with the aforementioned social and psychological explanations. The same genetic factors that influence marital conflict or parental disruptive behavior, might also affect child vulnerability for conduct problems (Harden et al. 2007). Previous studies revealed that parent-child resemblance for antisocial behavior is largely due to the genetic transmission of a general vulnerability to externalizing behavior (Ball et al. 2008; Hicks et al. 2013). Thus, our findings that a hostile or harsh father, or family distress predicts child bullying could be to a great extent the product of shared genes for aggressive traits rather than only a direct effect of social processes. However, when we assume genetic vulnerability as a main explanatory mechanism, fathers’ prenatal hostility and mothers’ hostility or harshness should both predict children’s bullying behaviors. Hence, genetic vulnerability may not be a sufficient explanation.

The social environment can reinforce inherited vulnerabilities such as aggressiveness. Not all children will be affected by exposure to an adverse environment, and genetic predispositions to aggressiveness may have different effects depending on the environment (Tuvblad and Baker 2011). Thus, children with an aggressive predisposition, who grow up in an adverse family environment in which the father is hostile and harsh, may display higher rates of bullying behavior than children with the same aggressive predisposition, who do not grow up in an adverse family environment. Our findings that not prenatal, but rather postnatal hostility of the father predicts child bullying behavior may suggest that next to any genetic predisposition to aggressive behavior, father-child interaction is essential to determine the negative impact of father’s hostility. This reasoning is in accordance with the findings of Jaffee et al. (2003) that children of fathers, who engage in antisocial behavior, have worse behavior problems if the father resides in the home.

Finally, there is a possibility of reverse causality that should be considered when explaining the association between family adversity and later child bullying behavior (e.g., child behavior problems can be a source of family distress, and disciplinary practices can be influenced by child behavior). However, we assessed parental hostility and family distress prior to the birth of the child, and thus the effects of these prenatal risk factors could not be influenced by child behavior. Moreover, in order to address reverse causality as an explanation of the significant associations between postnatal family adversity and child bullying behavior, we adjusted our analyses for externalizing behavioral problems at age 18 months. Even after this adjustment, hostility and harsh disciplinary practices remained predictors of children’s bullying behaviors. These findings suggest that it is unlikely that fathers react in a hostile or harsh manner because the child behaves difficult.

### Strengths and Limitations

Prior research on family adversity and children’s bullying behaviors has often failed to specifically include characteristics of both the father and the mother (e.g., Copeland et al. 2013; Schwartz et al. 1997; Burk et al. 2008). One of the major strengths of our study is that we utilized multiple informants (i.e., father and mother) to assess the risk factors, and multiple peer reports to assess the outcome. This strengthened the validity of the findings, and enabled us to study father’s and mother’s effects on child development separately. Another major strength of our study is its prospective longitudinal design, which is rare in studies of family adversity effects. Measuring parental hostility and family distress before the birth of the child and harsh parenting at preschool age, allowed us to examine the prospective development of the association. Finally, embedding our study in a large longitudinal cohort allowed us to adjust our analyses for numerous confounders.

Despite these strengths, our study has some limitations, which could be addressed in future studies. One limitation is that the non-response analyses indicated that some selection occurred toward families with a higher socioeconomic status. However, prospective cohorts (such as the Generation R Study) do not need to be representative of a population to be generalizable as long as the study population is sufficiently large and captures a diversity of exposures and backgrounds (Manolio and Collins 2010). Second, observational measurements of family adversity were not feasible in our large study. The self-reports that were used to assess family adversity could have led to socially desirable answers and to underestimation of the degree of family adversity. However, we used only questionnaires that were reliable and valid. Third, we excluded three items from the harsh disciplining questionnaire as they encompass illegal practices. This may reduce the comparability of the measure with other settings. Fourth, although the questionnaire measuring externalizing behaviors (CBCL/1½–5) is validated for 18-month-old children, some behaviors (e.g., “Cannot sit still”) might be normative for this age. However, we did not use this measure as an outcome, but only to assess whether the observed associations under study were independent of pre-existing externalizing problems. Fifth, in the current population-based sample, it was not possible to focus on families without a father.

### Implications

Previously, little attention has been given to the influence of fathers on the development of children’s bullying behaviors. Our findings highlight that the influence of fathers cannot be ignored. Future studies should collect information on fathers to further examine fathers’ risk factors that predispose children to engage in bullying behaviors.

Next to implications for future research, our findings have practical implications for anti-bullying interventions. School bullying prevention is an important public health goal (Srabstein and Leventhal 2010). Anti-bullying interventions can prevent serious outcomes that are directly related to school bullying, such as internalizing and externalizing problems (Ttofi and Farrington 2012). Early interventions should target children who display bullying behavior to prevent them from developing more serious forms of aggression and violence later in life (Sourander et al. 2007). However, anti-bullying interventions that focus solely on schools may not adequately address the source of child bullying behavior. As family adversity is an important predictor of children’s bullying behaviors, it is imperative to involve parents, in particular fathers, in anti-bullying interventions. For example, anti-bullying interventions could include parental training sessions to teach positive disciplinary practices, and to teach strategies on how to cope with negative or hostile feelings, and how to handle conflicts or stress within the family. Parent involvement in school-based anti-bullying interventions has indeed been linked to a reduction in bullying (Axford et al. 2015). If children who bully their peers are targeted as early as possible using interventions that involve the entire family, then this may ensure that the negativity emerging from family adversity is not spilled over to the child, and eventually result in a strong reduction of school bullying.

### Conclusion

From previous research, it is known that family adversity is associated with children’s bullying behaviors at school. The present study adds to this literature by demonstrating that father-reported family adversity is a risk factor for children’s bullying behaviors. In particular, although the effect sizes were relatively small, fathers’ hostile and harsh behaviors were related to children’s bullying behavior in elementary school, whereas the effect of the mothers’ behavior was less pronounced. An accurate understanding of how an adverse family environment predisposes children to engage in school bullying is key for future interventions.

## Electronic supplementary material

ESM 1 (DOCX 27.4 kb)

## Footnotes

1 Mother-reported family adversity also predicted children’s bullying behaviors over and above that of the background family risk factors, early childhood externalizing problems and father-reported family adversity (*χ* ^*2*^ (5) = 13.64, *p* < .05).
