# Supplementary material for: Like Father, like Child: Early Life Family Adversity and Children’s Bullying Behaviors in Elementary School
Source: J Abnorm Child Psychol. 2017 Dec 19;46(7):1481–96. doi: 10.1007/s10802-017-0380-8 (PMC6133006; doi:10.1007/s10802-017-0380-8)
Supplement: Supplementary file 1 — (DOCX 27.4 kb) [file 10802_2017_380_MOESM1_ESM.docx]

**Supplemental Material**

| Supplementary Table 1  *Background Family Risk Factor Items Per Domain* | |
| --- | --- |
| Domain | Individual items |
| Life stress | Child or partner died (yes) |
|  | Friend or relative died (yes) |
|  | Child or relative ill (yes) |
|  | Admitted to hospital > 24hrs during pregnancy (yes) |
|  | Health is moderate or poor (yes) |
|  | Become unemployed past year (yes) |
|  | Mother problems at work or school (yes) |
|  | *Father problems at work or school (yes)* |
|  | Moved house past year (yes) |
|  | Vaginal blood loss past 2 months (yes) |
|  | Chorionic villus sampling test, amniocentesis test, triple test, or ultrasound for nuchal translucency during pregnancy (yes) |
|  | Been a victim of robbery past year (yes) |
|  | Pregnancy was not planned (yes) |
|  | Almost always or often worried about health baby (yes) |
|  | Somewhat or very dissatisfied with obstetric care (yes) |
|  | Almost always or often worried about pregnancy (yes) |
|  |  |
| Contextual risks | Adequate heating, washing machine or refrigerator (no) |
|  | Cold, draft, misted windows, damp patches, mold, furniture problems (yes) |
|  | Slight, moderate or serious housing problems (yes) |
|  | Slight, moderate or serious financial problems (yes) |
|  | Some or great difficulty in paying food, rent or electricity (yes) |
|  | Downturn in financial situation past year (yes) |
|  |  |
| Other background factors | Criminal record mother (yes) |
|  | *Criminal record father (yes)* |
|  | Age mother during pregnancy < 19 years (yes) |
|  | Highest education is no education or primary (yes) |
|  | Mother ever been addicted (yes) |
|  | *Father ever been addicted (yes)* |
|  | Mother threatened, hit or injured someone 1 or more times (yes) |
|  | *Father threatened, hit or injured someone 1 or more times (yes)* |
|  | Mother deliberately damaged/vandalized something 1 or more times (yes) |
|  | *Father deliberately damaged/vandalized something 1 or more times (yes)* |

*Note.* Italic items are reported by the father.

| Supplementary Table 2  *Hierarchical Multilevel Model Predicting Children’s Bullying Behavior’s from Four Cumulative Blocks of Background Family Risk Factors, Early Childhood Behavior Problems, Mother-Reported Family Adversity, and Father-Reported Family Adversity (N = 1,298)* | | | | | | | | | | | | |
| --- | --- | --- | --- | --- | --- | --- | --- | --- | --- | --- | --- | --- |
|  | Block 1 | |  | Block 2 | |  | Block 3 | |  | Block 4 | |  |
| Variable | *B* | 95% CI |  | *B* | 95% CI |  | *B* | 95% CI |  | *B* | 95% CI | |
| Background family risk factors, prenatal | | | | | | | | | | | | |
| Life stress | 0.02 | [-0.03, 0.07] |  | 0.01 | [-0.04, 0.06] |  | 0.02 | [-0.04, 0.07] |  | 0.01 | [-0.05, 0.06] | |
| Contextual factors | 0.01 | [-0.04, 0.07] |  | 0.00 | [-0.05, 0.06] |  | 0.01 | [-0.05, 0.06] |  | 0.00 | [-0.06, 0.06] | |
| Other background factors | 0.12** | [0.04, 0.19] |  | 0.11** | [0.04, 0.19] |  | 0.12** | [0.04, 0.20] |  | 0.12* | [0.03, 0.21] | |
| Early childhood behavioral problems |  |  |  |  |  |  |  |  |  |  |  | |
| Externalizing problems 18 months |  |  |  | 0.05* | [0.00, 0.10] |  | 0.05 | [-0.01, 0.10] |  | 0.04 | [-0.02, 0.10] | |
| Mother-reported family adversity |  |  |  |  |  |  |  |  |  |  |  | |
| Prenatal hostility |  |  |  |  |  |  | -0.04 | [-0.10, 0.01] |  | -0.04 | [-0.10, 0.01] | |
| Prenatal family distress |  |  |  |  |  |  | 0.01 | [-0.05, 0.06] |  | -0.02 | [-0.08, 0.04] | |
| Hostility 3 years after birth |  |  |  |  |  |  | -0.02 | [-0.09, 0.04] |  | -0.03 | [-0.10, 0.04] | |
| Harsh discipline 3 years after birth |  |  |  |  |  |  | 0.00 | [-0.07, 0.07] |  | -0.02 | [-0.10, 0.06] | |
| Family distress 6 years after birth |  |  |  |  |  |  | 0.08** | [0.03, 0.13] |  | 0.07* | [0.02, 0.13] | |
| Father-reported family adversity |  |  |  |  |  |  |  |  |  |  |  | |
| Prenatal hostility |  |  |  |  |  |  |  |  |  | 0.01 | [-0.06, 0.09] | |
| Prenatal family distress |  |  |  |  |  |  |  |  |  | 0.06* | [0.002, 0.12] | |
| Hostility 3 years after birth |  |  |  |  |  |  |  |  |  | 0.03 | [-0.04, 0.09] | |
| Harsh discipline 3 years after birth |  |  |  |  |  |  |  |  |  | 0.05 | [-0.02, 0.11] | |
| *-2 log likelihood* | 3303.52 | |  | 3299.27 | |  | 3284.59 | |  | 3269.66 | |  |

*Note.* Presented coefficient: standardized *B* derived from multilevel linear regression analyses (using transformed variables). All models were adjusted for the following socio-demographic covariates: child gender, age, ethnicity, parity, and parental income. CI = confidence interval.
* *p* < .05. ** *p* < .01. *** *p* < .001.

| Supplementary Table 3  *Multilevel Regression Analyses Predicting Children’s Bullying Behavior’s from Background Family Risk Factors, Early Childhood Behavioral Problems and Family Adversity in a Subsample Excluding Single-Mother Families (n =1137)* | | | | | | | | | | | | |
| --- | --- | --- | --- | --- | --- | --- | --- | --- | --- | --- | --- | --- |
|  | | Bullying score | | | | | | | | | | |
|  | Unadjusted (Model 1) | | |  | Adjusted for socio-demographic covariates (Model 2) | |  | Additionally adjusted for background family risk factors and early childhood behavioral problems (Model 3) | | | |  |
| Variable | *B* | | 95% CI |  | *B* | 95% CI |  | *B* | 95% CI | |  |  |
| Background family risk factors, prenatal | | | | | | | | | |  |  |  |
| Life stress  Contextual factors  Other background factors | 0.05  0.09**  0.14** | | [-0.01, 0.10]  [0.04, 0.15]  [0.05, 0.23] |  | 0.03  0.05  0.12** | [-0.02, 0.09]  [-0.01, 0.11]  [0.04, 0.20] |  | n.a.  n.a.  n.a. |  | |  |  |
| Early childhood behavioral problems | | | | | | | | | |  |  |  |
| Externalizing problems 18 months | 0.10** | | [0.04, 0.16] |  | 0.06* | [0.01, 0.12] |  | n.a. |  | |  |  |
| Family adversity |  | |  |  |  |  |  |  |  | |  |  |
| Hostility, prenatal |  | |  |  |  |  |  |  |  | |  |  |
| Father  Mother | 0.06  0.04 | | [-0.02, 0.14]  [-0.02, 0.09] |  | 0.05  0.00 | [-0.02, 0.12]  [-0.05, 0.06] |  | 0.02  -0.04 | [-0.05, 0.09]  [-0.10, 0.03] | |  |  |
| Family distress, prenatal |  | |  |  |  |  |  |  |  | |  |  |
| Father  Mother | 0.14***  0.09** | | [0.09, 0.20]  [0.03, 0.14] |  | 0.11***  0.04 | [0.05, 0.16]  [-0.02, 0.09] |  | 0.08**  0.02 | [0.03, 0.14]  [-0.04, 0.07] | |  |  |
| Hostility, 3 years after birth | | | | | | | | | |  |  |  |
| Father  Mother | 0.07*  0.00 | | [0.02, 0.12]  [-0.06, 0.06] |  | 0.06*  0.00 | [0.01, 0.11]  [-0.06, 0.06] |  | 0.05  -0.02 | [0.00, 0.11]  [-0.09, 0.05] | |  |  |
| Harsh discipline, 3 years after birth |  |  |  |  |  |  |  |  |  |  |  |  |
| Father  Mother | 0.11***  0.08* | | [0.05, 0.17]  [0.02, 0.13] |  | 0.06*  0.03 | [0.01, 0.11]  [-0.03, 0.08] |  | 0.06*  0.00 | [0.00, 0.12]  [-0.06, 0.07] | |  |  |
| Family distress, 6 years after birth | | | | | | | | | |  |  |  |
| Mother | 0.12*** | | [0.07, 0.17] |  | 0.08** | [0.03, 0.13] |  | 0.06* | [0.01, 0.12] | |  |  |

*Note.* Presented coefficient: standardized *B* derived from multilevel linear regression analyses (using transformed variables). Model 2 was adjusted for the following socio-demographic covariates: child gender, age, ethnicity, parity, and parental income. Model 3 was adjusted for the socio-demographic covariates and additionally for background family risk factors (i.e., life stress, contextual risks, and other background factors) and early childhood behavioral problems (i.e., children’s externalizing problems at 18 months). CI = confidence interval.
* *p* < .05. ** *p* < .01. *** *p* < .001.
